# Evaluation of Traffic Density Parameters as an Indicator of Vehicle Emission-Related Near-Road Air Pollution: A Case Study with NEXUS Measurement Data on Black Carbon

**DOI:** 10.3390/ijerph14121581

**Published:** 2017-12-15

**Authors:** Shi V. Liu, Fu-Lin Chen, Jianping Xue

**Affiliations:** National Exposure Research Laboratory, Office of Research and Development, U. S. Environmental Protection Agency, Durham, NC 27711, USA; chen.fu-lin@epa.gov

**Keywords:** air pollutant, near-road, traffic density, vehicle emission, black carbon, exposure

## Abstract

An important factor in evaluating health risk of near-road air pollution is to accurately estimate the traffic-related vehicle emission of air pollutants. Inclusion of traffic parameters such as road length/area, distance to roads, and traffic volume/intensity into models such as land use regression (LUR) models has improved exposure estimation. To better understand the relationship between vehicle emissions and near-road air pollution, we evaluated three traffic density-based indices: Major-Road Density (MRD), All-Traffic Density (ATD) and Heavy-Traffic Density (HTD) which represent the proportions of major roads, major road with annual average daily traffic (AADT), and major road with commercial annual average daily traffic (CAADT) in a buffered area, respectively. We evaluated the potential of these indices as vehicle emission-specific near-road air pollutant indicators by analyzing their correlation with black carbon (BC), a marker for mobile source air pollutants, using measurement data obtained from the Near-road Exposures and Effects of Urban Air Pollutants Study (NEXUS). The average BC concentrations during a day showed variations consistent with changes in traffic volume which were classified into high, medium, and low for the morning rush hours, the evening rush hours, and the rest of the day, respectively. The average correlation coefficients between BC concentrations and MRD, ATD, and HTD, were 0.26, 0.18, and 0.48, respectively, as compared with −0.31 and 0.25 for two commonly used traffic indicators: nearest distance to a major road and total length of the major road. HTD, which includes only heavy-duty diesel vehicles in its traffic count, gives statistically significant correlation coefficients for all near-road distances (50, 100, 150, 200, 250, and 300 m) that were analyzed. Generalized linear model (GLM) analyses show that season, traffic volume, HTD, and distance from major roads are highly related to BC measurements. Our analyses indicate that traffic density parameters may be more specific indicators of near-road BC concentrations for health risk studies. HTD is the best index for reflecting near-road BC concentrations which are influenced mainly by the emissions of heavy-duty diesel engines.

## 1. Introduction

With increasing motor vehicle traffic and populations working and/or living in various near-road environments, exposure to traffic-related air pollutants has become an important factor in considering overall risk of air pollution for human health [1,2,3,4,5,6,7]. Compared with point sources of air pollution such as industrial operations, mobile sources of air pollution present additional challenges to measurements and are difficult to model [8]. 

Vehicle-released air pollutants include carbon monoxide (CO), nitrogen oxides (NO_x_), black carbon (BC), and particulate matter (PM) which contains some BC, benzene and other aromatic compounds such as polycyclic aromatic hydrocarbons (PAHs), as well as other pollutants [9]. BC is a relatively stable air pollutant as compared with other reactive air pollutants such as NO_x_ and more closely reflects air pollution from traffic activities because a majority of BC is contributed by emissions from vehicles, especially heavy-duty diesel vehicles (HDDVs) [10,11]. BC has been used as an indicator in studies on near-road air pollutant exposure and adverse health effects [7,12,13,14]. Thus, understanding the profiles of near-road BC may lead to the development of a traffic-specific air pollution indicator and comprehensive evaluation of health risks associated with exposure to traffic pollution [15]. Indeed, BC has been considered as a more specific indicator of the direct influence of local traffic-related particles, rather than total PM [16].

In the past some studies have specifically addressed the characteristics of BC concentrations in near-road environments such as city districts [17] or major highways [18]. For example, one study found elevated BC concentrations at schools adjacent to highways, and that the counts of trucks and buses, not cars, were associated with these increases [19]. Another study showed that both diesel idling and passing were statistically significant contributors to variability in BC concentrations [20]. Furthermore, it was found that BC-PAH association is related with proximity to emissions [21]. This association is reflected with a general observation of higher BC concentrations and higher BC/TOC (black carbon/total organic carbon) concentration ratios in urban soils as compared to rural soils, indicating fossil fuel combustion, especially from traffic emissions, as the main source of urban BC [22]. Fuel combustion has been estimated to represent 80 ± 6% of the BC emitted from China [23].

Median concentrations of BC generally decrease with increased distance from major roads and expected diesel traffic [10]. The number of diesel vehicles is an important predictor of personal exposure to BC by cyclists [24]. Conversely, driving on roads with low traffic intensities results in lower exposures than driving on roads with higher traffic intensities, indicating traffic intensity as a major explanatory variable for in-vehicle BC exposure [25]. BC concentrations near roads show diurnal variations strongly associated with vehicular traffic, peaking in rush hours in the morning and evening [26]; however, the shape of the peaks varied among different sites [11,27].

In spite of some field monitoring studies, there remains a lack of a reliable indicator to support cost-effective estimates of population exposure to traffic-related air pollutants. A study showed that low-income families were three times more likely to live within high traffic density (TD) areas than those with high income [28]. A follow-up study further showed that racial/ethnic and socioeconomic disparities exist on a national level in the U.S. with respect to lower-income and minority populations living near high traffic and road density areas [29]. In this study we evaluated three different traffic density indices: major road density (MRD), all-traffic density (ATD), and heavy-traffic density (HTD) for their potential as vehicle emission indicators. We calculated the correlations of these traffic density indices with BC concentrations measured in the Near-road Exposures and Effects of Urban Air Pollutants Study (NEXUS) [30]. The goal of this study was, without making extensive efforts to understand the complex dispersion of a near-road air pollutants as likely influenced by other factors such as meteorological and topographic factors, to find if any one or more of these different traffic indices can be used for predicting the near-road air pollutant contribution from vehicle emissions. We selected BC as the testing air pollutant because a preliminary analysis has shown some matches between modeled and observed BC concentrations in the studied areas, especially within the high-traffic (HT)/high diesel (HD) group [31]. The current analyses more convincingly show traffic density parameters may be useful indicators of near-road BC concentrations for health risk studies.

## 2. Materials and Methods

### 2.1. Measurement Sites and Black Carbon Data Collection

The NEXUS study [30,31] was designed to investigate the relationship between exposures to traffic-related air pollutants and respiratory health in children with asthma living near major roads in Detroit, MI, USA. The studied homes were selected based on the proximity of these homes to major roads with different amounts of traffic, particularly diesel traffic. As previously described, measurements of traffic-related air pollutants were collected during two field-sampling campaigns: the fall campaign from 25 September to 11 November 2010 and the spring campaign from 28 March to 4 May 2011 [30]. 

Continuous (1 min) BC measurements were collected using Magee Scientific microAeth Model AE51 aethalometers (Aethlabs, San Francisco, CA, USA) deployed at three stationary monitoring sites across the city that included two school areas and a site located 100 m north of an interstate highway (I-96). The same AE51 aethalometers were deployed outdoors in a subset of study participant homes (25 homes in the fall and 18 homes in the spring) with approximately half located within 150 m of a major road with annual average daily traffic (AADT) >90,000 total vehicles per day. The remaining homes were located in the same general neighborhood but farther away from the major roads (>300 m from roads with >25,000 AADT and >500 m from roads with >90,000 AADT). While BC measurements were collected each day at the three stationary sites, a different set of homes (i.e., different home locations) was monitored each week (approximately four homes/week) with a target of six days of monitoring for each home. The spring data included fewer homes because some children who participated in the earlier fall campaign dropped out in the next spring campaign. 

Data were downloaded from the aethalometers and filter tickets replaced daily, typically between 13:00 and 16:00 (Eastern Standard Time), depending on the sample location. To reduce instrument error, the first and last 15 min of each raw data file were removed, given evidence that the instrument signal was affected by the daily site visits. Five-minute BC concentrations were calculated from the change in attenuation in the raw 1 min instrument output over a fixed 5 min interval [32]. Hourly average concentrations were calculated from the 5 min concentration data when a minimum of 30 min of data collection occurred. 

### 2.2. Traffic Density Metrics

Besides using nearest distance to a major road and the total length of the major road as traffic-related parameters [33,34], we also evaluated three different traffic indices based on previous studies using traffic density for evaluating socioeconomic and racial differences in near-road exposure to air pollution [28,29]. We reasoned that these more refined indices may differentially reflect the various contributions of road density, traffic density, and vehicle type to the concentration variations in traffic-related air pollutants. The names and definitions of these indices are:

Major road density (MRD) = ∑ *L*/*A_B_*

All traffic density (ATD) = ∑ (*L* × *AADT*)/*A_B_*

Heavy traffic density (HTD) = ∑ (*L* × *CAADT*)/*A_B_*

where *L* and *A_B_*, followed a previous convention [28], represent length of major road and buffered area around the selected site, respectively. *AADT* represents annual average daily traffic and *CAADT* represents commercial annual average daily traffic. These annual averages (data from Michigan Department of Transportation (MDOT) at http://gis-mdot.opendata.arcgis.com/) were used for a correlation study on BC monitoring data collected in two different seasons, even though season-specific averages would be more appropriate if they were available. The units for MRD and for ATD and HTD are mi^−1^ and VMT/d/mi^2^, respectively, where VMT is defined as vehicle miles of travel [28].

The road/traffic density-based indices differ from road/traffic intensity-based indices such as those used in various land use regression (LUR) models [35,36] because they reflect the ratios of road area and traffic volume within a total area and also, in the case of HTD, a specific type of vehicles passing through the area. In other words, we believe that traffic density as defined as traffic intensity divided by road area is more informative and relevant than just the traffic intensity or road area alone. Differentiating vehicle types in traffic-related air pollution is important because some types of vehicle engines make more of a contribution to specific air pollutants. 

**Figure 1:**
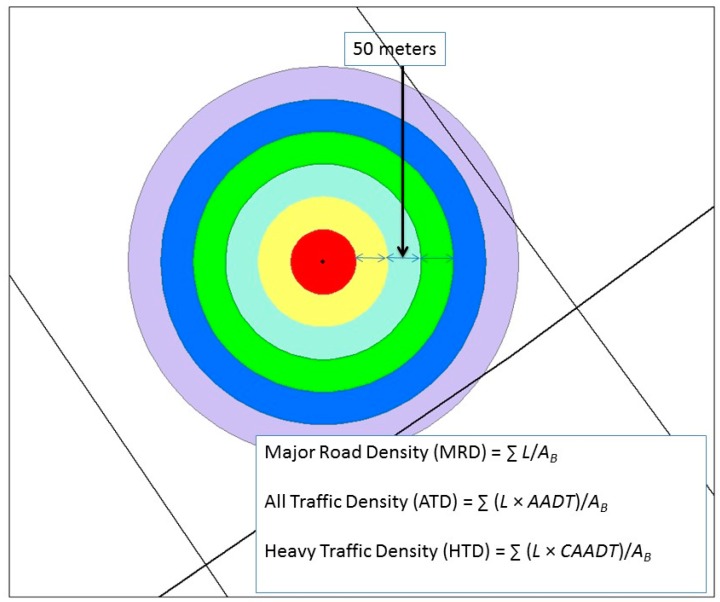
Concentric areas surrounding a participant’s home for calculating different traffic-related metrics. *L* and *A_B_* represent length of major road and buffered area around the selected site, respectively. *AADT* and *CAADT* represent annual average daily traffic and commercial annual average daily traffic, respectively.

Each participant’s home was treated as a center of multiple concentric circles with 50-m incremental spacing between rings for calculating traffic density within a different spatial extent around the home (see Figure 1). Sum of lengths or area of surface for all major roads with a 100-m buffer in each circle were calculated for major road density in each concentric circle of increasing sizes. 

### 2.3. Statistical Analyses

Correlation analyses were performed between each of three different traffic density indices and the averaged BC concentrations around studied homes and stationary monitoring sites (Figure S1). The general linear model (GLM), which incorporates a number of different statistical models into a generalization of multiple linear regression models to test a hypothesis in a multivariate or a univariate way, was used to show major factors affecting the correlation between BC concentrations and each traffic density index. Specifically, we performed several independent univariate tests to see if season, traffic volume, and heavy traffic density within a specific road distance range were correlated with BC concentrations.

## 3. Results

### 3.1. Change in Black Carbon Concentrations during a Day

**Figure 2:**
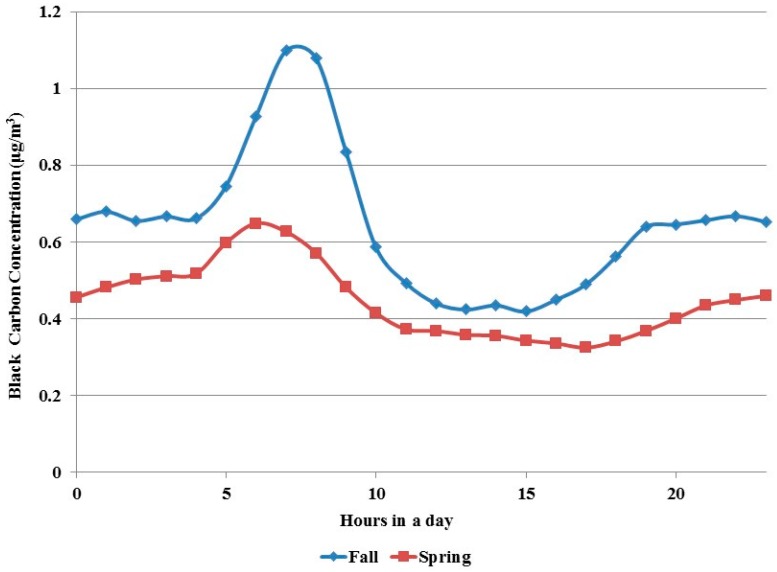
Annual average of diurnal concentrations of black carbon.

The hourly-averaged BC concentrations measured outside participants’ homes and stationary monitoring sites were pooled together to calculate general patterns of averaged daily BC concentrations by hour of the day. BC concentrations in Detroit during the fall and spring field campaigns showed a diurnal pattern (Figure 2). Higher concentrations were observed most often during morning rush hours between 05:00 and 10:00, forming a sharp peak around 07:00–08:00. The concentrations between 11:00 and 17:00 were the lowest. BC concentrations were moderately elevated during evening rush hours, with these levels persisting throughout the evening hours.

### 3.2. Statistics of BC Concentrations in the Two Monitoring Seasons

Because the average concentrations of BC in fall of 2010 were higher than those of spring 2011, for each corresponding time during a day we performed statistical analysis on the BC concentrations for each monitoring season with three levels of traffic volumes as shown in the diurnal patterns in Figure 1. The mean peak time (high traffic volume) BC concentration in the morning rush hours was 0.94 μg/m^3^ for the fall data and 0.59 μg/m^3^ for the spring data, respectively (Table 1). The off-peak time (low traffic volume) BC concentration was about 0.45 μg/m^3^ for the fall data and 0.35 μg/m^3^ for the spring data. The fall data showed larger difference (means ranging from 0.45 to 0.94 μg/m^3^ as compared with ranging from 0.35 to 0.59 μg/m^3^) and greater variability (standard deviation ranging from 0.44 to 0.92 μg/m^3^ as compared with ranging from 0.28 to 0.50 μg/m^3^) than the spring data in averaged BC concentration among three levels of traffic volume.

### 3.3. Correlation between Black Carbon Concentrations and Different Traffic Density Indices

To understand what traffic density indices are likely associated with changes in measured BC concentrations, in this NEXUS study we pooled both fall and spring measurement data together and performed a correlation analysis between BC concentrations and each of the three types of traffic density indices developed in this study as well as the two other previously used indices: nearest distance to major road (ND) and total length of major road (TL) (Table 2). The correlation between BC concentrations and the nearest distance to a major road showed a negative value of −0.31. The correlation between BC concentrations and the total length of a major road ranged between 0.15 and 0.32, with an average of 0.25. For major road density and traffic density index, the correlation coefficients between BC concentrations and traffic density decrease over increasing size of analyzed area; all correlation coefficients are not statistically significant. The average correlation coefficients for MRD and ATD were 0.26 and 0.18, respectively. The correlation coefficients for HTD were averaged at 0.48, which is a value much higher than those shown for other two indices. More importantly, the correlation coefficients between BC concentrations and HTD are statistically significant in every concentric-circled area around the study homes (Table 2).

### 3.4. Season-Separated Correlation Analysis between Black Carbon Concentrations and Traffic Density Indices

Because of the existence of some differences in BC concentrations between fall 2010 and spring 2011 (Figure 1) we performed a correlation analysis between BC concentrations and traffic density indices by separating the fall 2010 and the spring 2011 monitoring data. The result shows that the correlation coefficients were always higher and positive for the fall data but lower and some even negative for the spring data (Table 3). Interestingly, the high and statistically significant positive correlations between BC concentrations and HTD were seen only with the fall data but not with the spring data. The average correlation coefficients for HTD were about 0.53 for the fall data but only 0.1 for the spring data.

To see if traffic volume influenced the correlation between BC concentrations and traffic density index we performed additional analysis by adding another level of data segregation, i.e., by splitting BC concentration data into three levels of traffic volume: high in the morning rush hours, medium in the evening rush hours, and low for the rest of day. This analysis showed again that HTD was most significantly associated with BC concentrations in all fall measurements regardless of the difference in traffic volumes and size of concentric areas (Table S1). For the spring data, it is interesting to notice that some significant correlations were found between BC concentrations in low traffic volume hours for HTD as well as the other two traffic density indices.

### 3.5. General Linear Model Analysis on the Relationship between Black Carbon Concentrations and Traffic Metrics

The GLM analysis showed that season, traffic volume, and heavy traffic density were three major factors influencing BC concentrations (Table 4). Collectively, these three variables could explain 56% of the variance for the BC concentrations. It is interesting to note that the *F*-values for each 50-m concentric ring were different, with the 0–50 m range having the largest value of 16.57 and the 201–300 m range having the smallest value of 5.48.

## 4. Discussion

Similar to previous studies on BC concentrations near roads [11,26] a diurnal pattern of higher BC concentrations in rush hours was also observed in the studied Detroit area (Figure 1 and Table 1). This observation reinforces the conclusion that near-road BC concentration is related with the traffic volume or, in other words, intensity [23,24] which is also reflected in a report of air quality modeling for NEXUS data on BC [31]. In that modeling report the modeled and the observed BC concentrations were compared in three groups: HT (high traffic)/HD (high diesel), HT/LD (low diesel), and LT (low traffic). It seems that the HT/HD group generally showed better matches between the modeled and the observed BC concentrations than the other two groups, and the LT group showed the worst match. It was concluded that the discrepancy could be explained by the uncertainty in traffic activity at the road link level.

In comparing conventional traffic parameters such as ND and TL with our density-based traffic parameters, which are MRD, ATD and HTD, we found that, while MRD and ATD performed similarly to TL and all were better than ND, HTD performed best, showing the highest correlation coefficients that were all statistically more than significant (*p* < 0.05) in all near-road distance ranges (Table 2). This result is understandable as traffic-related BC release comes mainly from HDDVs which are counted for by CAADT specifically used in HTD. 

Although the pooled data showed a significant correlation between BC concentrations and HTD, the season-segregated analysis showed a significant correlation for the fall data but not for the spring data (Table 3). This apparent seasonal difference might be caused by the much lower BC concentrations in the spring than in the fall in the studied areas and/or the smaller sample size in the spring than the fall campaign. While studies with a larger sampling size and/or a collection of associated meteorological data may help to clarify this issue, it is really not essential for this study to sort out these potential complicating factors. This is because the focus of the current study was not on ascertaining if there was a true seasonal difference in BC concentration in this study site but on testing if there is a traffic density indicator which can be conveniently utilized for predicting vehicle emission contribution to near-road BC concentration.

As the homes selected for the NEXUS study are located near roads, increasing the size of concentric circle for calculating the road/traffic flowing by each nearby home might result in a “dilution” of traffic density in the enclosed area and thus some “reduction” in the correlation between traffic density indices and BC concentrations measured outside the near-road homes. This hypothesis may explain the general decrease of correlation coefficients between BC concentrations and major road density, as well as traffic density over increasing circular size (Table 2 and Table 3). 

However, with HTD, this trend appeared only for the spring data but not the fall data, possibly due to the much higher BC concentrations in the fall and thus a larger area of effect. Nevertheless, the GLM analysis (Table 4) showed highest *F*-value (16.57) for distances between 0–50 m from the monitored homes but the lowest value (5.48) for distances between 251–300 m from the monitored homes.

Thus, traffic density—the traffic intensity within a specific area—might be a better indicator than just road density or traffic intensity for reflecting local air concentration of vehicle emission-released pollutants. However, whether or not a use of traffic density-based indicator would improve land regression model of near-road air pollution [35,36] remains to be determined.

Because GLM analysis showed traffic volume/peak was also a factor influencing BC concentrations (Table 4), an analysis on correlations between BC concentrations and different traffic density indices was also performed, with segregation of both season and traffic volume/peak (Table S1). This detailed analysis showed additional significant associations even for the low traffic volume hours. For example, for both MRD and ATD, significant correlations were found for concentric circles at a distance of between 150 and 300 m. For HTD, the concentric circles between 50 and 150 m showed significant correlations.

A reason for this puzzling finding may be the higher percentage of heavy-duty diesel vehicles flowing through these regions during the hours without the commuting rush. Although we do not know the composition of vehicle types in the different time periods in the studied areas, one study has shown a diurnal pattern of the number of heavy-duty trucks in other areas peaking on weekdays during the middle of the day and falling off before the afternoon rush hour [37]. This might be a result of local restrictions on diesel traffic and/or a selection of a more free-flowing traffic time by the truck drivers. Thus, the low traffic volume hours might incidentally present a better chance to find some significant correlations between BC concentrations and the traffic density of heavy vehicles, not only for HTD but also the other two traffic density indices.

There are some limitations in this study that might also complicate the findings. For example, the traffic density indices are based on annual average values but traffic flow in the studied area might vary between seasons (spring vs. fall), days of the week (such as weekday vs. weekend), and hours (rush hours vs. non-rush hours). In addition, measurements of BC concentrations were made only in selected short periods and the homes monitored in the different time periods were not all the same. However, with a better understanding of the correlation between BC concentrations and newly developed traffic density indices, future studies with larger national air monitoring data and wide geographic locations with traffic density counts may help solidify the findings obtained in this study. Nevertheless, the general trends revealed for association of traffic density with black carbon concentration still provide some useful information for evaluating population exposure to traffic-related air pollutants and the related health risk.

## 5. Conclusions

Through correlation analysis of monitored BC concentrations with two conventional (nearest distance to major road and total length of major road) and three newly developed traffic parameters (MRD, ATD, and HTD), we found that BC concentrations in the studied areas were significantly correlated with HTD for all near-road distances (50, 100, 150, 200, 250, and 300 m) analyzed. These results suggest that HTD may serve as a useful index for urban air pollution resulting from traffic flow, especially the combustion emission of heavy-duty diesel engines. Future studies on other locations and different geographic sites with larger datasets may further refine the analysis and thus validate the value of using conveniently available traffic volume data with the newly developed traffic density indices for near-road air pollution exposure and health risk evaluation.

## Figures and Tables

**Table 1:** Simple statistics of black carbon concentrations (μg/m^3^).

Season	Traffic Volume *	*N*	Mean	SD	P5	P25	P50	P75	P95
Fall	Low	1657	0.45	0.44	0.07	0.17	0.31	0.60	1.22
	Medium	3200	0.64	0.63	0.07	0.19	0.49	0.93	1.72
	High	1317	0.94	0.92	0.10	0.29	0.65	1.31	2.71
Spring	Low	1147	0.35	0.28	0.08	0.18	0.29	0.42	0.84
	Medium	2181	0.44	0.36	0.09	0.21	0.35	0.56	1.10
	High	889	0.59	0.50	0.10	0.23	0.45	0.80	1.55

* Low: hours between 11:00 and 17:00; Medium: hours between 18:00 and 23:00 and 00:00 and 04:00; High: hours between 05:00 and 09:00. *N* means number of measurements; Means and SD are the average and standard deviation, respectively; and P5–P95 are the 5th–95th percentile.

**Table 2:** Correlation coefficients of traffic-related metrics with measured black carbon concentrations.

Traffic Parameter	Distance (m) from the Center of the Concentric Circles						Average
	50	100	150	200	250	300	
Nearest distance to a major road	−0.31						
Total length of a major road	0.30	0.32	0.28	0.23	0.20	0.15	0.25
Major road density	0.33	0.33	0.28	0.24	0.21	0.17	0.26
All-traffic density	0.25	0.26	0.21	0.16	0.12	0.09	0.18
Heavy traffic density	0.41 *	0.49 **	0.49 *	0.50 *	0.49 **	0.47 *	0.48

*p* < 0.05; ** *p* < 0.01.

**Table 3:** Season stratified correlation coefficients of traffic-related metrics with measured black carbon concentrations in two monitoring seasons.

Season	Distance (m) from the Center of the Concentric Circles						
	50	100	150	200	250	300	
	Major road density						
Fall	0.37	0.39 *	0.37	0.33	0.31	0.27	0.34
Spring	0.27	0.20	0.08	−0.01	−0.08	−0.15	0.05
	All-traffic density						
Fall	0.29	0.31	0.28	0.25	0.22	0.19	0.25
Spring	0.23	0.16	0.02	−0.08	−0.16	−0.22	−0.01
	Heavy traffic density						
Fall	0.47 **	0.54 **	0.55 **	0.55 **	0.55 **	0.54 **	0.53
Spring	0.24	0.21	0.14	0.07	0.00	−0.05	0.10

* *p* < 0.05; ** *p* < 0.01.

**Table 4:** General linear model (GLM) analysis on major factors affecting correlation between black carbon concentration and traffic density index.

Variable	Degree of Freedom	Type III SS	*F*-Value	Statistical Significance
Season	1	1.39	34.49	**
Traffic volume	2	3.20	39.71	**
Heavy traffic density within a distance range (m)				
0~50	1	0.67	16.57	**
51~100	1	0.36	8.98	**
101~200	1	0.47	11.76	**
201~300	1	0.22	5.48	*

SS: sums of squares; * *p* < 0.05; ** *p* < 0.01.

## Supplementary Materials

The following are available online at www.mdpi.com/1660-4601/14/12/1581/s1, Figure S1: Study site and monitoring locations, Table S1: Season- and traffic volume-stratified correlation coefficients of traffic-related metrics with measured black carbon concentrations in different near-road distances.
