# Supplementary material for: Evaluation of Traffic Density Parameters as an Indicator of Vehicle Emission-Related Near-Road Air Pollution: A Case Study with NEXUS Measurement Data on Black Carbon
Source: Int J Environ Res Public Health. 2017 Dec 15;14(12):1581. doi: 10.3390/ijerph14121581 (PMC5750999; doi:10.3390/ijerph14121581)
Supplement: Supplementary file 1 [file ijerph-14-01581-s001.pdf]

Supplemental Table 1. Season- and traffic volume-stratified correlation coefficients of traffic-related metrics with measured black carbon concentrations in different near-road distances.

Season	Traffic volume	Distance (meter) off the center of the concentric circles						
		50	100	150	200	250	300	Average
		Major road density						
Fall	Low	0.30	0.37	0.40*	0.40*	0.41*	0.40*	0.38
	Medium	0.35	0.36	0.33	0.29	0.25	0.21	0.30
	High	0.37	0.37	0.33	0.28	0.25	0.21	0.30
Spring	Low	0.55**	0.46*	0.35	0.25	0.16	0.09	0.31
	Medium	0.17	0.10	-0.03	-0.11	-0.18	-0.25	-0.05
	High	0.16	0.12	0.03	-0.03	-0.08	-0.13	0.01
		All traffic density						
Fall	Low	0.27	0.36	0.39*	0.40*	0.41*	0.40*	0.37
	Medium	0.24	0.26	0.22	0.17	0.13	0.10	0.19
	High	0.30	0.29	0.25	0.20	0.16	0.13	0.22
Spring	Low	0.59**	0.49*	0.36	0.24	0.14	0.07	0.31
	Medium	0.11	0.04	-0.10	-0.21	-0.28	-0.33	-0.13
	High	0.12	0.09	-0.02	-0.08	-0.14	-0.18	-0.04
		Heavy traffic density						
Fall	Low	0.38**	0.50**	0.57**	0.62**	0.65**	0.66**	0.56
	Medium	0.42**	0.47*	0.47*	0.46*	0.43*	0.43*	0.45
	High	0.51**	0.55**	0.54**	0.53**	0.49**	0.49**	0.52
Spring	Low	0.61**	0.57**	0.50*	0.43	0.31	0.31	0.46
	Medium	0.15	0.12	0.04	-0.04	-0.16	-0.16	0.00
	High	0.07	0.06	0.01	-0.03	-0.11	-0.11	-0.01

* $P < 0.05$; ** $P < 0.01$

Supplementary Figure 1.

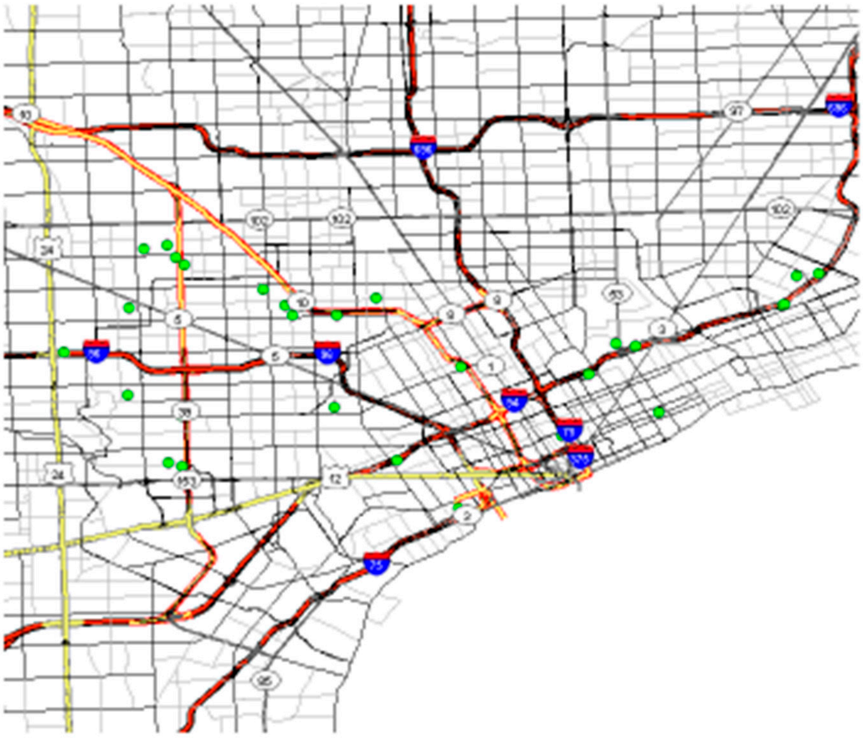

Supplemental Figure 1. Study site and monitoring locations.
